# The Role of the TSK/TONSL-H3.1 Pathway in Maintaining Genome Stability in Multicellular Eukaryotes

**DOI:** 10.3390/ijms23169029

**Published:** 2022-08-12

**Authors:** Yi-Chun Huang, Wenxin Yuan, Yannick Jacob

**Affiliations:** Department of Molecular, Cellular and Developmental Biology, Faculty of Arts and Sciences, Yale University, 260 Whitney Avenue, New Haven, CT 06511, USA

**Keywords:** DNA repair, DNA replication, histone H3.1 variant, homologous recombination, genome stability

## Abstract

Replication-dependent histone H3.1 and replication-independent histone H3.3 are nearly identical proteins in most multicellular eukaryotes. The N-terminal tails of these H3 variants, where the majority of histone post-translational modifications are made, typically differ by only one amino acid. Despite extensive sequence similarity with H3.3, the H3.1 variant has been hypothesized to play unique roles in cells, as it is specifically expressed and inserted into chromatin during DNA replication. However, identifying a function that is unique to H3.1 during replication has remained elusive. In this review, we discuss recent findings regarding the involvement of the H3.1 variant in regulating the TSK/TONSL-mediated resolution of stalled or broken replication forks. Uncovering this new function for the H3.1 variant has been made possible by the identification of the first proteins containing domains that can selectively bind or modify the H3.1 variant. The functional characterization of H3-variant-specific readers and writers reveals another layer of chromatin-based information regulating transcription, DNA replication, and DNA repair.

## 1. Introduction

Histones are the core protein components of chromatin. An octamer containing two copies of histones H2A, H2B, H3, and H4 organizes approximately 147 bp of DNA into a nucleosome, which forms the basic structural unit of chromatin [1]. Although nucleosomes are always composed of the same four classes of histones, multiple non-allelic isoforms, or histone variants, were described, primarily in the H2A, H2B, and H3 protein families [2,3,4]. These variants can be conserved over extensive evolutionary distances, or specific to certain phylogenetic clades [5].

Amino acid differences between histone variants of the same family have long been proposed to have functional implications. The H3 barcode hypothesis put forward by Hake and Allis in 2006 formalized this idea by proposing that H3 variants could be subjected to distinct post-translational signatures. In this framework, the enrichment of a particular H3 variant at specific regions of the genome would define epigenomic domains and determine the chromatin-based functions at these loci [6]. Testing this hypothesis has been challenging, in part due to the high level of similarity between some histone variants. For example, the two main non-centromeric H3 variants of multicellular eukaryotes, H3.1 and H3.3, differ by only one (e.g., in animals) or two (e.g., in vascular plants) amino acids in their N-terminal unstructured tail. The observed sequence similarity brings the very important question of whether a few amino acid differences between H3.1 and H3.3 can be sufficient to regulate the catalytic activity of histone-modifying enzymes or the binding of histone readers.

Over the past 10 years, histone-variant-specific readers and writers have started to be identified, thus confirming the direct roles of histone variants in regulating chromatin-based activities, including transcription, DNA replication, and DNA repair. These initial findings may represent the tip of the iceberg in terms of how histone variants function in cells to shape different epigenetic states in eukaryotic systems. In order to incite further investigations of histone variants, this review highlights the main findings from the past few decades of research on the conserved H3.1 variant. Recent advances in elucidating a specific role for H3.1 in promoting replication fork progression are also discussed.

## 2. Discovery of the Replication-Dependent H3.1 Variant

**Figure 1:**
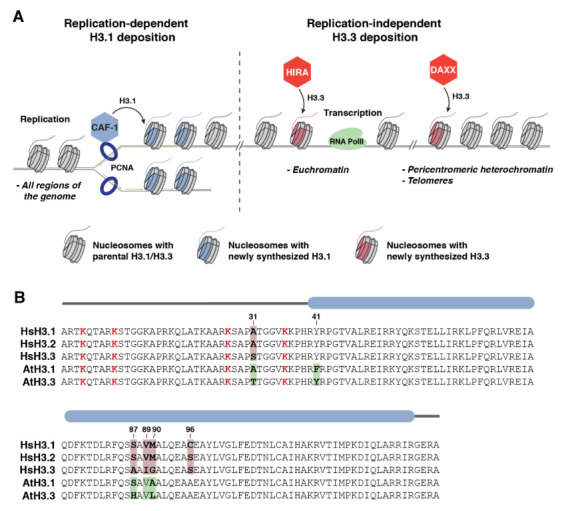
The distinct deposition pathways and sequences of histone H3.1 and H3.3 variants: (**A**) The deposition of H3.1 and H3.3 by their dedicated histone chaperones at different genomic loci. The CAF-1 complex directly interacts with the PCNA clamp and deposits H3.1 at replication forks during DNA replication. In contrast, HIRA inserts H3.3 at active gene regions (i.e., euchromatin) during transcription, while DAXX deposits H3.3 mainly at various heterochromatic loci, including pericentromeric regions and telomeres. Nucleosomes with parental H3.1 or H3.3 histones are shown in gray; nucleosomes with newly synthesized H3 proteins are highlighted in blue (H3.1) or red (H3.3); (**B**) sequence alignment of histone H3 variants (H3.1/H3.2 and H3.3) from human and Arabidopsis. The sequence folding into the globular domain of H3 is marked by a blue rounded rectangle. Functionally important lysine residues (i.e., K4, K9, K27, and K36) in the N-terminal tails of H3 variants are highlighted in red. Sequence variations between H3.1, H3.2, and H3.3 in humans and Arabidopsis are indicated in pink and green, respectively, with positional information for these variable amino acids indicated above the alignment.

The H3.1 variant was first identified in 1977 by Franklin and Zweindler, who used gel electrophoresis to distinguish different histone proteins extracted from mammalian tissues [7]. Many years after, it was shown that, in multicellular eukaryotes, H3.1 is inserted into chromatin in a replication-dependent manner (Figure 1A) [8,9]. The specific use of H3.1 during replication has led this histone variant to be named the replication-dependent histone H3 variant. A few years later, it was shown that H3.1 can also be inserted into chromatin in response to UV irradiation at damaged genomic loci in human cells [10]. The other major H3 protein, the non-centromeric histone H3.3 variant, is inserted into chromatin in a replication-independent manner (Figure 1A) [8,9]. Comparative analyses suggested that the H3.1 variant evolved from the more ancestral H3.3 [11]. The differential insertion of H3 variants into chromatin is regulated at the transcriptional level, with the expression of H3.1 peaking specifically during DNA replication, while H3.3 is constitutively expressed throughout the cell cycle [12,13]. In addition, the deposition of H3.1 and H3.3 onto chromatin is mediated by distinct histone chaperones [14]. The heterotrimeric CAF-1 complex loads H3.1 at replication forks to maintain nucleosome density genome-wide during replication (Figure 1A) [15]. CAF-1 can directly interact with the sliding clamp proliferating cell nuclear antigen (PCNA), which contributes to H3.1 incorporation [16]. In contrast, the H3.3 variant is inserted into chromatin by two other histone chaperones: HIRA and DAXX (Figure 1A) [15,17,18,19]. HIRA mediates the replacement of H3.1 with H3.3 during transcription, a process facilitated by the interaction of HIRA with many factors involved in RNA synthesis [20,21,22]. As a result, H3.3 is mostly found in transcriptionally active regions of the genome in proliferative cells [18]. However, H3.3 is also present in several regulatory regions of the genome [18] and genome-wide in post-mitotic cells [23,24,25,26] and the chromatin of reproductive cells in Drosophila *(Drosophila melanogaster*), mouse (*Mus musculus*), and Arabidopsis (*Arabidopsis thaliana*) [27,28,29,30]. Studies on the histone chaperone DAXX have shown that it deposits H3.3 at the telomeres and specific regions of the pericentromeric heterochromatin in embryonic stem (ES) cells [17,18]. In proliferative cells, the active replacement of H3.1 with H3.3 at specific loci results in a genome-wide enrichment of the H3.1 variant in heterochromatic, transcriptionally silent regions of the genome [31,32].

One of the most striking aspects of the H3.1 and H3.3 variants is how similar they are at the primary sequence level. In vascular plants, only 4 out of 135 amino acids (positions 31, 41, 87, and 90) vary between H3.1 and H3.3 (Figure 1B) [12]. Research focusing on the rDNA arrays of Arabidopsis determined that amino acids 87 and 90 of H3.3 regulate its deposition at active loci, while residues 31 and 41 guide nucleosome disassembly after rDNA transcription is interrupted [33]. Another study in Arabidopsis demonstrated that the H3.1-specific residue phenylalanine 41 (Phe41 or F41) plays an important role in regulating the genome-wide distribution of H3.1 [34]. In animals, H3.1 and H3.3 also differ at four positions (31, 87, 89, and 90). Variations at amino acids 87 and 90 were shown to be required for the variant-specific interaction of H3.3 with its histone chaperones HIRA and DAXX [35,36,37], thus providing a molecular mechanism for the differential mode of deposition of H3 variants onto chromatin. Interestingly, mammals (but not other metazoans) contain another replication-dependent H3 variant, H3.2, which differs from H3.1 in only one position (S96 instead of C96) (Figure 1B). The effect on chromatin of variation at position 96 of H3 is not well-understood.

Most post-translational modifications (PTMs) deposited on histones occur in their unstructured N-terminal tails [38]. As only a single amino acid residue (position 31) conservatively varies between H3.1 and H3.3 in the tail of all eukaryotes (Figure 1B), it had been unclear whether H3.1 and H3.3 variants could be marked by distinct PTMs, and if so, what would be the underlying mechanisms. Initial studies identified differential patterns of PTMs on H3 variants [39,40,41,42,43,44]. These patterns typically showed that active marks were enriched in H3.3, while repressive marks were predominantly located on H3.1. These observations of H3-variant-specific PTMs raised the question of whether H3 variants could have a direct impact on the deposition of these PTMs. An alternative explanation would be that the H3-variant-specific patterns of PTMs are simply a consequence of the enrichment of particular H3 variants in different regions of the genome. To test these two hypotheses, which are not mutually exclusive, studies were initiated to identify histone writers that could selectively modify H3.1 or H3.3. In addition to potentially regulating histone H3 writers, other molecular mechanisms could be mediated by H3.1 and H3.3 to provide functional specificity to chromatin loci containing these H3 variants. For example, differential binding by histone readers to a histone PTM present on H3.1 and H3.3 could provide a way to generate functional differences for H3.1 and H3.3. Similarly, amino acid variations between H3.1 and H3.3 could be sufficient (without the associated PTMs) to directly affect H3 binding by histone readers. For all of these reasons, finding H3-variant-specific readers and writers became an important endeavor in the field of chromatin biology.

## 3. Identification of Readers and Writers of H3 Variants

In searching for evidence about the specificity of H3 variants in regard to the deposition of histone PTMs, phosphorylation at serine 31 (S31ph) of H3.3 was a logical modification to investigate first, as non-phosphorylatable alanine replaces serine/threonine at position 31 in the H3.1 variant (Figure 1B). Earlier studies have shown that different regions of the genome contain H3.3S31ph during mitosis and meiosis, although these regions vary by cell type and organism [45,46,47,48]. Currently, the precise role of H3.3S31ph at these stages of the cell cycle remains unclear. More recent studies have demonstrated a role for H3.3S31ph in gastrulation [49], embryonic stem cell differentiation [50], and the regulation of stimulated-gene expression [51]. H3.3S31ph has been shown or proposed to regulate binding to H3.3, by either favoring or preventing the interaction of the reader proteins JMJD3/KDM6B and PHF1 [51,52]. Symmetrically, the absence of phosphorylation at H3.3S31 can also direct variant-specific interactions, as the transcriptional regulator ZMYND11 binds H3K36me3 specifically on H3.3 by reading unmodified S31 [53,54].

In regard to H3.1-specific readers and writers, aside from the early discoveries that identified CAF-1 as being the H3.1 chaperone, most of the new findings have come from studies in the model plant Arabidopsis. The H3K27 monomethyltransferases ATXR5 and ATXR6 (ATXR5/6), which are represented in all land plants and *Tetrahymena thermophila* [55,56] but are absent in animals, were the first histone-modifying enzymes shown to specifically methylate the H3.1 variant [56]. A recent follow-up study related to ATXR5/6 has revealed a universal H3.1 reader domain in the protein TONSOKU (TSK) (known as TONSOKU-LIKE/TONSL in animals), which participates in the regulation of DNA repair during replication [57]. The discoveries of proteins that can write or read H3.1 are presented in greater depth in the next sections.

### 3.1. H3.1-Specific Deposition of H3K27me1 by ATXR5 and ATXR6 in Plants

ATXR5/6 were initially characterized as SET-domain proteins capable of directly interacting with PCNA, a central regulator of replication and DNA repair [58], via a conserved PCNA-interacting protein (PIP) motif [59]. In the same study, *ATXR6* was shown to be expressed at the transition between the G_1_ and the S phase of the cell cycle, while *ATXR5* is constitutively expressed in cycling cells. A comparative analysis of all SET-domain proteins in Arabidopsis initially classified ATXR5/6 as H3K4 methyltransferases [60], but a subsequent study established that they are in fact part of a distinct class of SET-domain proteins conserved in land plants [61]. In vitro and in vivo enzymatic assays demonstrated that ATXR5/6 monomethylate H3K27 (H3K27me1) [62]. This finding thus defined ATXR5/6 as a new class of H3K27 methyltransferases in plants, in addition to the one formed by the widely conserved PRC2 enzymes [63]. The loss of H3K27me1 in *atxr5 atxr6* (*atxr5/6*) double mutants results in the transcriptional derepression of repetitive sequences and heterochromatin decondensation [62], which is in line with the enrichment of H3K27me1 in transcriptionally silent regions of the genome [64,65]. Additional work identified a role for ATXR5/6 in maintaining genome stability in plants, as *atxr5/6* mutants show a genomic amplification of heterochromatic sequences (hereafter referred to as heterochromatin amplification) [64]. The role of ATXR5/6 in preserving heterochromatin stability and silencing was shown to be dependent on their interaction with PCNA and their catalytic SET domain, thus indicating that the deposition of H3K27me1 during replication is critical to maintaining genomic stability in plants [64].

**Figure 2:**
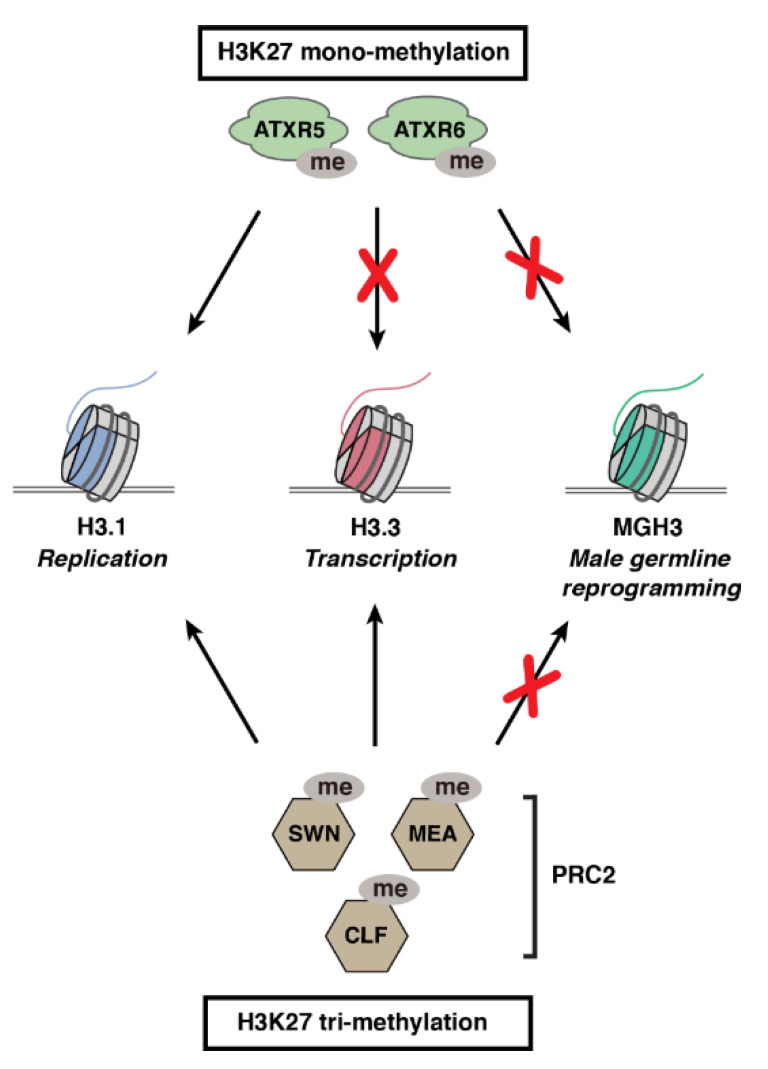
Activity of plant H3K27 methyltransferases on different H3 variants. ATXR5 and ATXR6 catalyze K27 monomethylation on H3.1 variants. PRC2 complexes containing either CLF, MEA, or SWN (catalytic subunits of Arabidopsis PRC2 complexes) trimethylate K27 on H3.1 and H3.3 variants. In contrast, ATXR5, ATXR6, and PRC2 complexes cannot methylate the male-gamete-specific histone variant MGH3, which contributes to reprograming the paternal epigenome in pollen of Arabidopsis.

While trying to understand the molecular role played by H3K27me1 in safeguarding against heterochromatin defects, it was found that the K27 monomethyltransferase activity of ATXR5/6 is specific for the H3.1 variant (H3.1K27me1) (Figure 2) [56]. The selective methylation of histone H3 variants by different histone methyltransferases was tested based on the idea that sequence variation between different histone variants near a target substrate (e.g., K27) may promote or interfere with enzymatic activity. This idea was initially assessed for H3.1 and H3.3 at position K27 due to the nearby variation at position 31 between these H3 variants (Figure 1B), and results showed that ATXR5/6 were in fact only active on the H3.1 variant. In contrast, different PRC2 complexes containing the catalytic subunits CURLY LEAF (CLF), SWINGER (SWN), or MEDEA (MEA) can equally methylate K27 on H3.1 and H3.3 variants in vitro (Figure 2) [56] (Y. Jacob, unpublished data). More recent work has shown that CLF, MEA, and ATXR5/6 are incapable of methylating the male-gamete-specific histone H3.3-like variant MGH3 (also known as HTR10) in Arabidopsis, with functional consequences for germ cell reprogramming (Figure 2) [66]. Even though MGH3 contains a K27 residue, this variant differs from the canonical H3.3 by a stretch of ~5 highly divergent amino acids located immediately downstream of K27 [12]. The functional characterization of H3K27 methyltransferases from Arabidopsis demonstrates that the modulation of chromatin writer activity by H3 variants can regulate different cellular processes in plants.

Structural work revealed that the H3.1 specificity of ATXR5/6 is conferred by their conserved nSET region, which folds into a cage-like structure around the canonical SET domain [56]. The nSET-SET arrangement generates a selectivity pocket in the catalytic domain of ATXR5/6 that can accommodate residue A31 of H3.1, but not T31 of plant H3.3 variants. Plants expressing H3.1 variants with alanine 31 replaced with threonine (H3.1A31T) displayed a loss of H3.1K27me1 in vivo [56]. Interestingly, ATXR5/6 are able to methylate a mutant H3.1 variant containing serine at position 31 as efficiently as the wild-type H3.1 [67]. The fact that T31, but not S31, can inhibit ATXR5/6 activity provides an explanation as to why plant H3.3 variants differ from their mammalian orthologs at this position (Figure 1B). The ATXR5/6 ortholog in *T. thermophila* (i.e., TXR1) does not appear to preferentially methylate H3.1 over H3.3 [68], and this observation is in line with the fact that both H3.1 and H3.3 variants participate in replication-coupled histone deposition in that organism [69].

The specificity of ATXR5/6 for H3.1 in plants suggested a unique role for this H3 variant in maintaining genome stability and transcriptional silencing. However, it was unclear whether or not such a role for H3.1 would be conserved in animals. The recent identification of the DNA repair protein TSK/TONSL as an H3.1 reader protein provides new insights into these questions [57].

### 3.2. TSK/TONSL Acts as an H3.1 Reader via its TPR Domain

**Figure 3:**
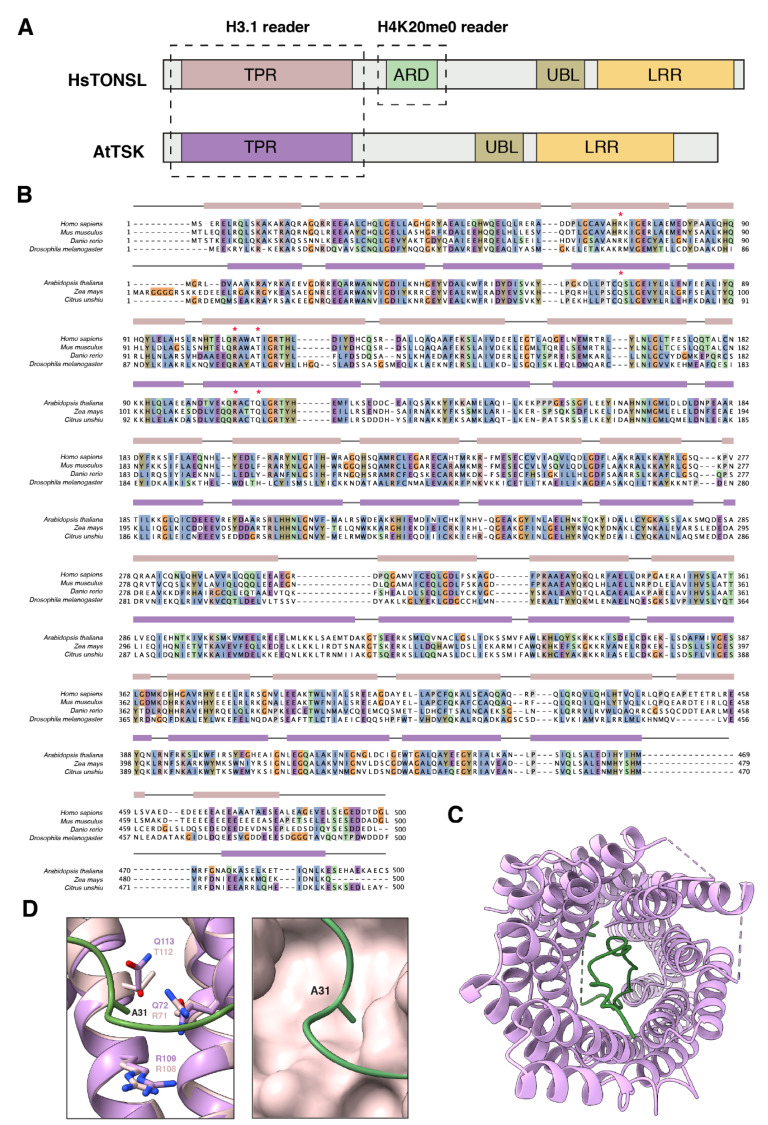
The TPR domain of TSK/TONSL functions as an H3.1 reader: (**A**) Domain architecture of human and Arabidopsis TONSL/TSK. TPR: tetratricopeptide repeats, ARD: ankyrin repeat domain, UBL: ubiquitin-like, LRR: leucine-rich repeats. The domains in TSK/TONSL responsible for binding specific histones are marked by dashed rectangles; (**B**) alignment of TPR domains from multiple animal and plant TSK/TONSL orthologs. NCBI reference sequences: NP_038460.4 (*Homo sapiens*), NP_898914.3 (*Mus musculus*), NP_001104618.1 (*Danio rerio*), Q9VSA4.1 (*Drosophila melanogaster*), NP_188503.2 (*Arabidopsis thaliana*), PWZ29356.1 (*Zea mays*), and GAY58445.1 (*Citrus unshiu*). Residues in the alignment are colored according to the Clustal X color scheme. The α helices of the animal and plant TPR domains of TONSL/TSK, respectively, shown as pink or violet rectangles above the alignment, were predicted by AlphaFold (animals) or based on the crystal structure of the TPR domain of *C. unshiu* TSK (plants). Red asterisks indicate the residues of the TPR domains shown (plants) or predicted (animals) to mediate specific binding to H3.1 via recognition of residue A31; (**C**) top view of the TPR domain of TSK (violet) from *C. unshiu* co-crystalized with the histone H3.1 N-terminal tail (green) (PDB accession number: 7T7T); (**D**) (Left panel) structural superposition of the solved TPR domain (violet) of plant TSK (*C. unshiu*) and the AlphaFold-predicted TPR domain (pink) of TONSL (human), with a focus on the histone H3.1A31-binding pocket. The amino acid residues from the TPR domain of plant TSK or animal TONSL interacting with H3.1A31 (green) are shown. (Right panel) Surface representation of the predicted H3.1A31-binding pocket from the TPR domain of human TONSL.

TONSOKU (TSK), also known as BRUSHY1 (BRU1) or MGOUN3 (MGO3), was initially characterized in plants as a putative DNA repair protein, as *tsk* mutants were shown to be hypersensitive to genotoxic stress [70]. In addition, other cellular and developmental phenotypes associated with *tsk* mutants, including fasciation, disorganized shoot, and root apical meristem, stochastic loss of gene silencing, and altered heterochromatin organization, suggested a possible role for *TSK* in the establishment and/or maintenance of epigenetic states [70,71,72]. A few years later, multiple studies revealed that the mammalian ortholog of TSK, known as TONSOKU-LIKE (TONSL), plays a critical role in repairing broken forks via homologous recombination during DNA replication [73,74,75,76] (see next section). TONSL was demonstrated to associate with soluble non-nucleosomal H3/H4 heterodimers in vivo [73,77], and further work identified a direct interaction between full-length TONSL and H3 peptides in vitro [77]. In addition, mammalian TONSL was shown to directly interact with H4K20me0 via its ankyrin repeat domain (ARD), which plays an essential role in recruiting TONSL to chromatin to mediate DNA repair during replication (Figure 3A) [78]. Interestingly, the ARD domain is absent from the plant TSK proteins [57,73], thus suggesting that TSK and TONSL are recruited to chromatin through different mechanisms.

The identification of TSK/TONSL as DNA repair proteins rescuing broken replication forks, the reported interaction between TONSL and histone H3, and the role of ATXR5/6 in maintaining genome stability in plants via H3.1K27me1 during replication all pointed to a possible link between these proteins. The functional relationship between H3.1, ATXR5/6, and TSK/TONSL was confirmed in a recent study that showed that TSK acts as an H3.1 reader protein via a conserved tetratricopeptide repeat (TPR) domain [57]. The TPR domain in TSK is composed of 11 repeated TPR motifs (hairpins of antiparallel α-helices) that fold into a channel-like solenoid (Figure 3B) [57]. The N-terminal tail of the H3.1 variant (amino acids 1–40) fits in this domain by folding back inside the structure (Figure 3C). A small binding pocket, strictly conserved across plant TSK orthologs, selectively reads the unique residue A31 of H3.1 [57]. Importantly, the TPR domain of mammalian TONSL also displays a preference for binding the H3.1 variant over H3.3 in vitro [57]. The AlphaFold model for the TPR domain of TONSL predicts a similar organization of the α-helices forming the interacting pocket with H3.1A31 compared with plants [79] (Figure 3D). Interestingly, there are variations in some of the main residues contributing to reading A31 between plants and animals (Figure 3B,D). This observation may reflect the need to differentiate against H3.3S31 in animals, as this residue is replaced with T31 in plant H3.3 (Figure 1B). Additional structural studies are needed to fully understand the similarities and differences in the way plant TSK and animal TONSL selectively interacts with H3.1.

Overall, these studies identified the first H3.1-specific reader domain, which appears to be universally conserved in multicellular eukaryotes. The specificity of both ATXR5/6 and TSK for the H3.1 variant suggests that these proteins are part of a common pathway in plants. The functional characterization of TONSL as a mediator of replication-coupled DNA repair in mammals suggests a similar function for TSK in plants.

## 4. Role of the TSK/TONSL-H3.1 Pathway in DNA Repair

Most of the work on the role of TSK/TONSL in DNA repair has been performed in mammalian cells. TONSL has been shown to interact with multiple proteins, including ASF1, MCM2/4/5/6/7, and most importantly, MMS22L [73,74,75,76,77]. TONSL and MMS22L form a stable complex in vivo, which is mediated by the C-terminal LRR domain of TONSL (Figure 3A) [73,75]. Depleting the mammalian cells of TONSL or MMS22L generates similar phenotypes, such as hypersensitivity to the genotoxic drug camptothecin, activation of ATR-dependent signaling, the formation of 53BP1/γH2AX/phospho-RPA-positive foci, a delay in inactivating DNA damage response (DDR) signaling after DNA damage, and defects in homologous recombination (HR) [73,74,75,76]. TONSL–MMS22L has been shown to colocalize with RPA and the histone H2A.X variant in vivo, and initial results indicated a role for the complex in facilitating the loading of the recombinase RAD51 [73,75]. Further experiments showed that, while the TONSL–MMS22L complex is present at unperturbed replication forks, it is enriched at collapsed forks containing resected DNA [80]. MMS22L was shown to directly interact with RPA-coated ssDNA, and also with RAD51, which directly links the HR repair machinery to the TONSL–MMS22L complex [80]. In vitro and in vivo experiments suggested that TONSL can rescue perturbed replication forks via two different mechanisms relying on RAD51: replication fork reversal and the homologous recombination-mediated repair of dsDNA breaks [80]. Interestingly, although the TONSL–MMS22L complex directly binds to RAD51 and contributes to RAD51-mediated strand exchange required for HR in vitro, it does not catalyze the assembly of RAD51 filament on ssDNA coated with RPA. These findings suggest the presence of additional, unknown proteins working with TONSL–MMS22L [80].

**Figure 4:**
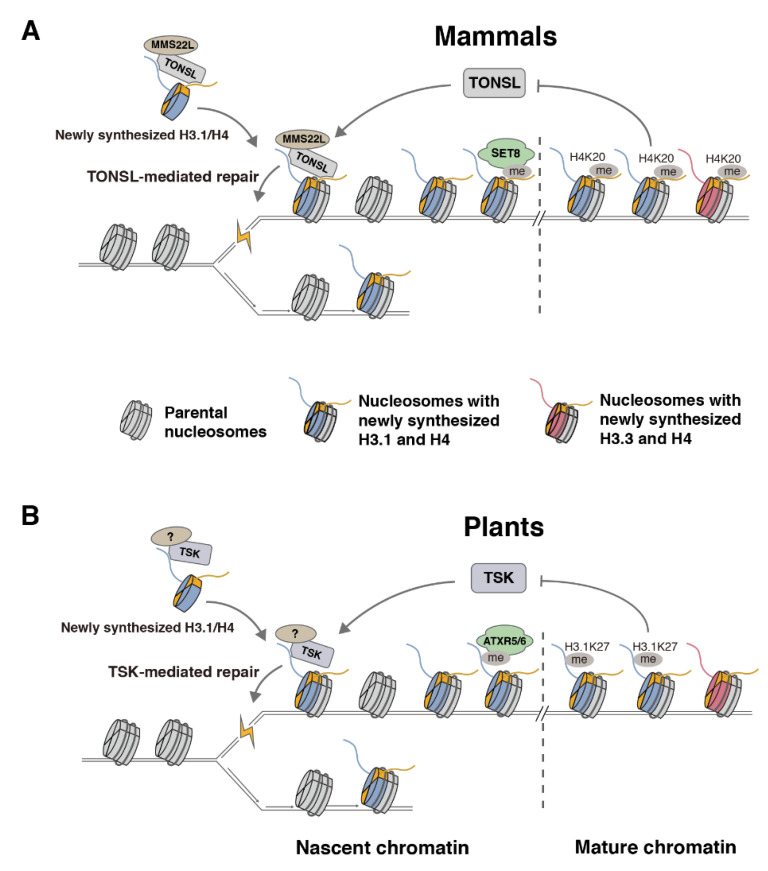
Comparison of the chromatin-based mechanisms regulating TSK/TONSL activity between mammals and plants: (**A**) In mammals, TONSL forms a heterodimer with MMS22L and initially interacts with soluble H3.1/H4 using its TPR and ARD domains. After H3.1/H4 incorporation into nascent chromatin, TONSL–MMS22L recruits HR repair proteins to resolve stalled or broken replication forks. Post-replicative chromatin maturation relies on SET8 to monomethylate H4K20, which prevents the binding of TONSL–MMS22L to mature chromatin; (**B**) plants lack a clear MMS22L homologous protein, and TSK only contains a TPR domain to mediate the interaction with newly synthesized H3.1/H4. In addition, H3.1K27, not H4K20, is monomethylated (via ATXR5/6) to prevent TSK from interacting with mature chromatin. Parental H3/H4 histones are shown in gray; newly synthesized histone proteins are highlighted in blue (H3.1), red (H3.3) or yellow (H4).

A major leap in our understanding of the regulatory mechanisms affecting TONSL activity came from the discovery that its ARD domain mediates the interaction with H4K20me0 [78]. In vitro and in vivo experiments demonstrated that the recruitment of TONSL to chromatin, and its DNA repair activity, are dependent on the ARD domain [78]. In animals, H4K20me0 is a signature of post-replicative chromatin, which lasts until late G2 during the cell cycle [78]. The maturation of post-replicative chromatin is then accomplished via SET8/PR-Set7/SETD8, which monomethylates H4K20 (H4K20me1) and, thus, blocks TONSL from binding chromatin [78]. The regulation of TONSL via SET8 ensures that TONSL activity is temporally and spatially restricted. Based on these findings and previous work, the authors proposed a model where TONSL is delivered during S phase to nascent chromatin in a pre-deposition complex with ASF1, MCM2, and H3/H4 tetramers (Figure 4A). The replication-dependent insertion of TONSL promotes its localization at replication forks and post-replicative chromatin, which facilitates DNA repair via HR at broken forks. One question resulting from the discovery that TONSL interacts with H4K20me0 was whether the TONSL–MMS22L complex can also be recruited to chromatin when H3.3/H4 complexes are deposited by the HIRA and DAXX chaperones. There is no evidence suggesting that H4 is specifically methylated at K20 before the replication-independent insertion of H3.3/H4 to prevent the recruitment of TONSL. However, the recent discovery that TSK/TONSL specifically interacts with H3.1 via its TPR domain provides a mechanism to shuffle TSK/TONSL away from H3.3/H4 and replication-independent histone deposition [57]. A study using human cell lines has shown that TONSL activity requires the H3.1 chaperone CAF-1, thus supporting a conserved role in multicellular eukaryotes for the H3.1 variant in promoting TSK/TONSL activity [81].

Comparative functional analyses in plant and animal systems suggest a similar strategy for regulating TSK/TONSL activity and post-replicative chromatin maturation via histone methylation (Figure 4B). The absence of a SET8 ortholog in plants, combined with the lack of an ARD domain in plant TSK (Figure 3A) [73], can be explained by the activity of the plant-specific enzymes ATXR5/6, which appear to provide an analogous function to SET8 in monomethylating post-replicative chromatin to prevent the recruitment of TSK [57]. Genetic experiments in *atxr5/6* mutants or in plants expressing H3.1S28A (which prevents ATXR5/6-catalyzed H3.1K27me1 [82]) showed that heterochromatin amplification associated with the loss of H3K27me1 is dependent on H3.1 and TSK, thus suggesting that H3.1K27me0 positively regulates TSK activity in plants [57]. In addition, plants that express H3.1A31T (alanine 31 replaced with threonine, as in plant H3.3 variants) are hypersensitive to the replication blocking agent methyl methanesulfonate (MMS) [57], which mimics the phenotypes of *tsk* and *h3.1* mutants growing in the presence of this chemical [70]. Taken together, these results reveal the importance of ATXR5/6-mediated H3.1K27me1 in repressing TSK and suggest that plants and animals employ similar mechanisms to regulate TSK/TONSL-mediated DNA repair and post-replicative chromatin maturation. The precise regulation of TSK/TONSL appears to be critical in multicellular eukaryotes, as genomic instability occurs when this DNA repair pathway is lost or extended outside of its normal boundaries mediated by histone monomethylation from ATXR5/6 or SET8 [64,70,73,74,75,76,83,84,85,86].

## 5. Differences between Plants and Animals in the TSK/TONSL-H3.1 Pathway

In spite of many similarities between plants and animals in regulating the TSK/TONSL activity and post-replicative chromatin maturation, important mechanistic differences should still be considered. One striking difference between plant and animal systems is the chromatin target used for regulating the TSK/TONSL binding to chromatin. In plants, ATXR5/6 carry this function by selectively monomethylating H3.1K27 (Figure 4B) [56,57,62]. In animals, they rely on SET8 to monomethylate H4K20 (Figure 4A) [78,87,88,89]. This difference explains the presence of the ARD domain in TONSL, and its absence in plant TSK [73]. It also justifies why SET8 orthologs are absent in plants, and why ATXR5/6 orthologs are not found in animals. These observations are in line with other DNA replication or repair proteins that depend on H4K20 for their activity in animals [90,91,92] but not in plants, as the orthologous proteins lack H4K20-binding domains. More work is needed to understand the relative contributions of the TPR domain and the ARD domain of TONSL in mediating the interaction with H3.1/H4 tetramers during chromatin replication. The crystal structure of the ARD domain of TONSL with the H3/H4 tetramer could only be obtained by creating an artificial fusion protein containing the ARD domain of TONSL and the H3-binding domain of MCM2 [78]. The TPR domain of TONSL may, therefore, be required to allow for stable H3.1/H4 binding in vivo. However, the coexistence of both TPR and ARD domains in TONSL also raises the possibility that the TONSL interaction with H3.1/H4 may be differentially regulated, for example, in various cells/tissues, stages of the cell cycle, and/or regions of the genome.

Another area to explore for assessing possible differences between the TSK/TONSL-H3.1 pathway in animals and plants is with regard to post-translational modifications on H3.1 potentially regulating the DNA repair activity of TONSL. A study using mammalian HeLa S3 cells showed that PRC2 complexes are recruited to nascent chromatin right after the passage of the replication fork [93]. Furthermore, in vitro binding assays have shown that the interaction of full-length TONSL with H3.1 peptides is sensitive to mono-, di-, or trimethylation at lysine 27 [77]. Therefore, although no ATXR5/6 ortholog exists in animals [63], it is possible that the PRC2-mediated H3K27 methylation participates, together with SET8-catalyzed H4K20me1, in post-replicative chromatin maturation to prevent TONSL activity in animals. Similar to plants, most nucleosomal H3 proteins are methylated at K27 in animals, with H3K27me2 being the most abundant methylated state [94]. The high levels of K27 methylation on chromatin-bound H3 proteins suggest that unmethylated H3.1K27 variants may only be available at a specific moment during the cell cycle (i.e., at replicating DNA) for the recruitment of TONSL to chromatin.

Experiments in plants have shown that H3.1K27me1 levels do not appear to decrease during DNA replication, which has been explained by the fact that ATXR5/6 are able to rapidly methylate newly incorporated H3.1 variants as they are recruited at replication forks via their direct interaction with PCNA [95]. This may indicate that the presence of TSK on chromatin is restricted to regions near replication forks in plants. In contrast, TONSL has been shown to remain bound to chromatin until the late G_2_ phase of the cell cycle in mammals [78], which coincides with the monomethylation of K20 in new H4 proteins [78,89,96]. Although both plant ATXR5/6 and animal SET8 directly interact with PCNA [59,84,97], this interaction has been demonstrated to promote histone monomethylation in Arabidopsis [64] but to prevent it in mammalian cells via S-phase-specific, CLR4(CDT2)-mediated SET8 degradation [98,99,100,101,102]. However, there are discrepancies regarding the effect of PCNA on ATXR5/6 activity in plants, as in vitro data show that the interaction of ATXR5/6 with PCNA interferes with H3.1K27 monomethylation [103]. Therefore, more work is needed to resolve the potential differences between plants and animals regarding (1) the role of PCNA in regulating histone methylation during replication, and (2) how long TSK/TONSL can promote DNA repair during the cell cycle.

The actual DNA repair mechanism mediated by TSK/TONSL may also vary between plants and animals. In mammals, the MMS22L protein acts as a bridge between TONSL and RAD51 and is, therefore, required for repairing dsDNA breaks during replication via homologous recombination [73,74,75,76,80]. Although the C-terminal LRR domain of TONSL responsible for binding MMS22L is conserved in plant TSK (Figure 3A) [73,75], no MMS22L ortholog appears to be encoded in plant genomes. Experiments in Arabidopsis showed that two different DNA repair pathways are dependent on TSK: homologous recombination and polymerase theta-mediated end joining (TMEJ) [57]. These experiments were conducted in the *atxr5/6* mutant background, which artificially increases the TSK activity due to lower levels of H3.1K27me1. One possible mechanism to explain all these results would be for TSK in plants to directly promote DNA resection, an early step in DNA repair that is shared by both the HR and TMEJ pathways [104]. In line with this hypothesis, *RAD17*, which loads the MRE11–RAD50–NBS1 complex responsible for DNA resection in eukaryotes [105], was shown to be required for TMEJ-dependent heterochromatin amplification in *atxr5/6* mutants and HR-mediated repair in wild-type Arabidopsis [106].

## 6. Conclusions and Perspectives

The functional characterization of ATXR5/6 and TSK in plants has revealed many new insights regarding the conserved H3.1 variant. The identification of the first protein domains acting as an H3.1 reader (the TPR domain of TSK/TONSL) and writer (the SET domain of ATXR5/6) should prompt researchers to look for other proteins capable of selectively interacting with or modifying H3.1 or other histone variants. Aside from the canonical H3.1 and H3.3 proteins, many lineage-specific H3 variants have yet to be fully characterized. For example, plants contain many H3.3-like variants (e.g., HTR6, HTR10, HTR14, and HTR15 in Arabidopsis) that are defined by amino acid variations (compared with the canonical H3.3) located near modifiable residues [12,107]. Similarly, the human genome encodes four other H3 variants (H3.4, H3.5, H3.X, and H3.Y) in addition to H3.1, H3.2, H3.3, and CenH3 [108]. The activity of certain histone readers and/or writers may be altered in the cells where these lineage-specific H3 variants are expressed, with consequences in terms of chromatin regulation. In regard to the canonical H3.1 and H3.3, much of the work in plants has focused so far on the impact of variable position 31 on K27 readers and writers. However, the modifiable residue K36 is also in close proximity to position 31 and, therefore, represents a strong candidate for mediating differential activity on H3.1 and H3.3, as observed already in animals with ZMYND11 specifically binding H3.3K36me3 [53,54]. In addition, H3.1 and H3.3 vary at position 41 in vascular plants [34], and one study has shown that this residue can provide H3K36 specificity for the activity of a histone-modifying enzyme [109]. In terms of histone-variant-reading domains, it will be interesting to investigate if TPR domains in other proteins also have the ability to interact with histones and, more specifically, with histone variants. Thus far, the only other reports of a TPR domain interacting with histones are the ones from the histone chaperone sNASP/HIF1 [110,111], which interacts with the globular domain of different histone H3 variants (i.e., H3.1, H3.3, and CenH3). The presence of a TPR domain in other proteins associated with chromatin-related functions (e.g., UTX and SMYD3) suggests that this domain mediates the interaction of various proteins with histones [112,113].

Aside from investigating histone variants, many interesting questions remain to be addressed regarding the TSK/TONSL-H3.1 pathway. For example, identifying the binding partners of TSK will be essential to unravel the mechanism(s) used to resolve impaired replication forks in plants. Assuming additional similarity in how this DNA repair pathway operates in plants and animals, the functional characterization of the TSK pathway in plants should contribute to a better understanding of TONSL-mediated DNA repair in animals, and the etiology of human diseases linked to mutations in TONSL [114,115,116]. The identification of the numerous genetic suppressors of heterochromatin amplification in *atxr5/6* mutants provides candidate proteins that may work closely with TSK to preserve genome integrity [82,117,118,119,120], although multiple different mechanisms are likely involved in suppressing amplification in this mutant background [121]. Another important research direction will be to try to understand the developmental phenotypes associated with the loss of TSK/TONSL. In Arabidopsis, *tsk* mutants generate developmental phenotypes that are associated with epigenetic changes at key loci [122]. It will be important to determine if these alterations are linked to defects in genome stability or other functions of TSK during chromatin replication.

The discovery that H3.1 can selectively regulate TSK/TONSL recruitment to chromatin suggests that other proteins may similarly rely on H3.1 to regulate their activity during the replicative phase of the cell cycle. Mutation or the transcriptional modulation of the genes coding for these H3.1-interacting proteins could, therefore, directly affect other important processes occurring at the time of chromatin replication, which, if disrupted, may lead to diseases characterized by replication-dependent genomic and/or epigenomic instability. Disease-associated genomic instability phenotypes may also arise from mutations in genes that code for proteins involved in the deposition of H3.1 variants on chromatin. For example, genome instability characterized by the loss of rDNA repeats and large tandem genic duplications was recently observed in Arabidopsis plants containing a mutation in the H3.1 chaperone CAF-1 [123], and many human malignancies have been characterized as containing mutations in the subunits of this histone chaperone [124]. In addition, the discovery of specific roles for H3.1 and H3.3 suggests that mutations in histone H3 genes may generate distinct phenotypes depending on which specific H3 variant is mutated. H3-variant-specific effects seem to be the case for the well-studied H3K27M oncohistone mutation associated with pediatric diffuse midline gliomas [125,126], which completely eliminate H3K27me3 if the mutation is present on H3.1 but only reduce H3K27me3 levels when the mutation is located on H3.3 [127].

Clearly, more work lies ahead if we want to fully understand all the roles played by the H3.1 variant, or any other histone variant, in regulating chromatin-based processes in multicellular eukaryotes. However, the discovery of H3.1-variant-specific readers and writers able to recognize even the slightest molecular determinant (e.g., a single amino acid variation) to regulate their activity demonstrates that every amino acid matters in histones and that sequence variation between closely related histone variants likely underpins many functions in chromatin.

## References

[1] Luger K., Mader A.W., Richmond R.K., Sargent D.F., Richmond T.J. (1997). Crystal structure of the nucleosome core particle at 2.8 A resolution. Nature.

[2] Henikoff S., Smith M.M. (2015). Histone variants and epigenetics. Cold Spring Harb. Perspect. Biol..

[3] Jiang D., Borg M., Lorkovic Z.J., Montgomery S.A., Osakabe A., Yelagandula R., Axelsson E., Berger F. (2020). The evolution and functional divergence of the histone H2B family in plants. PLoS Genet..

[4] Talbert P.B., Henikoff S. (2021). Histone variants at a glance. J. Cell Sci..

[5] Talbert P.B., Ahmad K., Almouzni G., Ausio J., Berger F., Bhalla P.L., Bonner W.M., Cande W.Z., Chadwick B.P., Chan S.W. (2012). A unified phylogeny-based nomenclature for histone variants. Epigenetics. Chromatin..

[6] Hake S.B., Allis C.D. (2006). Histone H3 variants and their potential role in indexing mammalian genomes: The “H3 barcode hypothesis”. Proc. Natl. Acad. Sci. USA.

[7] Franklin S.G., Zweidler A. (1977). Non-allelic variants of histones 2a, 2b and 3 in mammals. Nature.

[8] Ahmad K., Henikoff S. (2002). The histone variant H3.3 marks active chromatin by replication-independent nucleosome assembly. Mol. Cell.

[9] Ray-Gallet D., Woolfe A., Vassias I., Pellentz C., Lacoste N., Puri A., Schultz D.C., Pchelintsev N.A., Adams P.D., Jansen L.E. (2011). Dynamics of histone H3 deposition in vivo reveal a nucleosome gap-filling mechanism for H3.3 to maintain chromatin integrity. Mol. Cell.

[10] Polo S.E., Roche D., Almouzni G. (2006). New histone incorporation marks sites of UV repair in human cells. Cell.

[11] Postberg J., Forcob S., Chang W.J., Lipps H.J. (2010). The evolutionary history of histone H3 suggests a deep eukaryotic root of chromatin modifying mechanisms. BMC Evol. Biol..

[12] Okada T., Endo M., Singh M.B., Bhalla P.L. (2005). Analysis of the histone H3 gene family in Arabidopsis and identification of the male-gamete-specific variant AtMGH3. Plant J..

[13] Wu R.S., Bonner W.M. (1981). Separation of basal histone synthesis from S-phase histone synthesis in dividing cells. Cell.

[14] Ray-Gallet D., Almouzni G. (2021). The Histone H3 Family and Its Deposition Pathways. Adv. Exp. Med. Biol..

[15] Tagami H., Ray-Gallet D., Almouzni G., Nakatani Y. (2004). Histone H3.1 and H3.3 complexes mediate nucleosome assembly pathways dependent or independent of DNA synthesis. Cell.

[16] Shibahara K., Stillman B. (1999). Replication-dependent marking of DNA by PCNA facilitates CAF-1-coupled inheritance of chromatin. Cell.

[17] Drane P., Ouararhni K., Depaux A., Shuaib M., Hamiche A. (2010). The death-associated protein DAXX is a novel histone chaperone involved in the replication-independent deposition of H3.3. Genes. Dev..

[18] Goldberg A.D., Banaszynski L.A., Noh K.M., Lewis P.W., Elsaesser S.J., Stadler S., Dewell S., Law M., Guo X., Li X. (2010). Distinct factors control histone variant H3.3 localization at specific genomic regions. Cell.

[19] Ray-Gallet D., Quivy J.P., Scamps C., Martini E.M., Lipinski M., Almouzni G. (2002). HIRA is critical for a nucleosome assembly pathway independent of DNA synthesis. Mol. Cell.

[20] Sarai N., Nimura K., Tamura T., Kanno T., Patel M.C., Heightman T.D., Ura K., Ozato K. (2013). WHSC1 links transcription elongation to HIRA-mediated histone H3.3 deposition. Embo. J..

[21] Soni S., Pchelintsev N., Adams P.D., Bieker J.J. (2014). Transcription factor EKLF (KLF1) recruitment of the histone chaperone HIRA is essential for beta-globin gene expression. Proc. Natl. Acad. Sci. USA.

[22] Zhang H., Gan H., Wang Z., Lee J.H., Zhou H., Ordog T., Wold M.S., Ljungman M., Zhang Z. (2017). RPA Interacts with HIRA and Regulates H3.3 Deposition at Gene Regulatory Elements in Mammalian Cells. Mol. Cell.

[23] Maze I., Wenderski W., Noh K.M., Bagot R.C., Tzavaras N., Purushothaman I., Elsasser S.J., Guo Y., Ionete C., Hurd Y.L. (2015). Critical Role of Histone Turnover in Neuronal Transcription and Plasticity. Neuron.

[24] Pina B., Suau P. (1987). Changes in histones H2A and H3 variant composition in differentiating and mature rat brain cortical neurons. Dev. Biol..

[25] Rogakou E.P., Sekeri-Pataryas K.E. (1999). Histone variants of H2A and H3 families are regulated during in vitro aging in the same manner as during differentiation. Exp. Gerontol..

[26] Urban M.K., Zweidler A. (1983). Changes in nucleosomal core histone variants during chicken development and maturation. Dev. Biol..

[27] Ingouff M., Rademacher S., Holec S., Soljic L., Xin N., Readshaw A., Foo S.H., Lahouze B., Sprunck S., Berger F. (2010). Zygotic resetting of the HISTONE 3 variant repertoire participates in epigenetic reprogramming in Arabidopsis. Curr. Biol..

[28] Loppin B., Bonnefoy E., Anselme C., Laurencon A., Karr T.L., Couble P. (2005). The histone H3.3 chaperone HIRA is essential for chromatin assembly in the male pronucleus. Nature.

[29] Torres-Padilla M.E., Bannister A.J., Hurd P.J., Kouzarides T., Zernicka-Goetz M. (2006). Dynamic distribution of the replacement histone variant H3.3 in the mouse oocyte and preimplantation embryos. Int. J. Dev. Biol..

[30] Van der Heijden G.W., Dieker J.W., Derijck A.A., Muller S., Berden J.H., Braat D.D., van der Vlag J., de Boer P. (2005). Asymmetry in histone H3 variants and lysine methylation between paternal and maternal chromatin of the early mouse zygote. Mech. Dev..

[31] Stroud H., Otero S., Desvoyes B., Ramirez-Parra E., Jacobsen S.E., Gutierrez C. (2012). Genome-wide analysis of histone H3.1 and H3.3 variants in *Arabidopsis thaliana*. Proc. Natl. Acad. Sci. USA.

[32] Wollmann H., Holec S., Alden K., Clarke N.D., Jacques P.E., Berger F. (2012). Dynamic deposition of histone variant h3.3 accompanies developmental remodeling of the Arabidopsis transcriptome. PLoS Genet..

[33] Shi L., Wang J., Hong F., Spector D.L., Fang Y. (2011). Four amino acids guide the assembly or disassembly of Arabidopsis histone H3.3-containing nucleosomes. Proc. Natl. Acad. Sci. USA.

[34] Lu L., Chen X., Qian S., Zhong X. (2018). The plant-specific histone residue Phe41 is important for genome-wide H3.1 distribution. Nat. Commun..

[35] Elsasser S.J., Huang H., Lewis P.W., Chin J.W., Allis C.D., Patel D.J. (2012). DAXX envelops a histone H3.3-H4 dimer for H3.3-specific recognition. Nature.

[36] Liu C.P., Xiong C., Wang M., Yu Z., Yang N., Chen P., Zhang Z., Li G., Xu R.M. (2012). Structure of the variant histone H3.3-H4 heterodimer in complex with its chaperone DAXX. Nat. Struct. Mol. Biol..

[37] Ricketts M.D., Frederick B., Hoff H., Tang Y., Schultz D.C., Singh Rai T., Grazia Vizioli M., Adams P.D., Marmorstein R. (2015). Ubinuclein-1 confers histone H3.3-specific-binding by the HIRA histone chaperone complex. Nat. Commun..

[38] Kouzarides T. (2007). Chromatin modifications and their function. Cell.

[39] Benson L.J., Gu Y., Yakovleva T., Tong K., Barrows C., Strack C.L., Cook R.G., Mizzen C.A., Annunziato A.T. (2006). Modifications of H3 and H4 during chromatin replication, nucleosome assembly, and histone exchange. J. Biol. Chem..

[40] Hake S.B., Garcia B.A., Duncan E.M., Kauer M., Dellaire G., Shabanowitz J., Bazett-Jones D.P., Allis C.D., Hunt D.F. (2006). Expression patterns and post-translational modifications associated with mammalian histone H3 variants. J. Biol. Chem..

[41] Johnson L., Mollah S., Garcia B.A., Muratore T.L., Shabanowitz J., Hunt D.F., Jacobsen S.E. (2004). Mass spectrometry analysis of Arabidopsis histone H3 reveals distinct combinations of post-translational modifications. Nucleic. Acids. Res..

[42] Loyola A., Bonaldi T., Roche D., Imhof A., Almouzni G. (2006). PTMs on H3 variants before chromatin assembly potentiate their final epigenetic state. Mol. Cell.

[43] McKittrick E., Gafken P.R., Ahmad K., Henikoff S. (2004). Histone H3.3 is enriched in covalent modifications associated with active chromatin. Proc. Natl. Acad. Sci. USA.

[44] Waterborg J.H. (1990). Sequence analysis of acetylation and methylation in two histone H3 variants of alfalfa. J. Biol. Chem..

[45] Hake S.B., Garcia B.A., Kauer M., Baker S.P., Shabanowitz J., Hunt D.F., Allis C.D. (2005). Serine 31 phosphorylation of histone variant H3.3 is specific to regions bordering centromeres in metaphase chromosomes. Proc. Natl. Acad. Sci. USA.

[46] Schulmeister A., Schmid M., Thompson E.M. (2007). Phosphorylation of the histone H3.3 variant in mitosis and meiosis of the urochordate Oikopleura dioica. Chromosome. Res..

[47] Van der Heijden G.W., Derijck A.A., Posfai E., Giele M., Pelczar P., Ramos L., Wansink D.G., van der Vlag J., Peters A.H., de Boer P. (2007). Chromosome-wide nucleosome replacement and H3.3 incorporation during mammalian meiotic sex chromosome inactivation. Nat. Genet..

[48] Wong L.H., Ren H., Williams E., McGhie J., Ahn S., Sim M., Tam A., Earle E., Anderson M.A., Mann J. (2009). Histone H3.3 incorporation provides a unique and functionally essential telomeric chromatin in embryonic stem cells. Genome. Res..

[49] Sitbon D., Boyarchuk E., Dingli F., Loew D., Almouzni G. (2020). Histone variant H3.3 residue S31 is essential for Xenopus gastrulation regardless of the deposition pathway. Nat. Commun..

[50] Martire S., Gogate A.A., Whitmill A., Tafessu A., Nguyen J., Teng Y.C., Tastemel M., Banaszynski L.A. (2019). Phosphorylation of histone H3.3 at serine 31 promotes p300 activity and enhancer acetylation. Nat. Genet..

[51] Armache A., Yang S., Martinez de Paz A., Robbins L.E., Durmaz C., Cheong J.Q., Ravishankar A., Daman A.W., Ahimovic D.J., Klevorn T. (2020). Histone H3.3 phosphorylation amplifies stimulation-induced transcription. Nature.

[52] Andrews F.H., Gatchalian J., Krajewski K., Strahl B.D., Kutateladze T.G. (2016). Regulation of Methyllysine Readers through Phosphorylation. ACS Chem. Biol..

[53] Guo R., Zheng L., Park J.W., Lv R., Chen H., Jiao F., Xu W., Mu S., Wen H., Qiu J. (2014). BS69/ZMYND11 reads and connects histone H3.3 lysine 36 trimethylation-decorated chromatin to regulated pre-mRNA processing. Mol. Cell.

[54] Wen H., Li Y., Xi Y., Jiang S., Stratton S., Peng D., Tanaka K., Ren Y., Xia Z., Wu J. (2014). ZMYND11 links histone H3.3K36me3 to transcription elongation and tumour suppression. Nature.

[55] Gao S., Xiong J., Zhang C., Berquist B.R., Yang R., Zhao M., Molascon A.J., Kwiatkowski S.Y., Yuan D., Qin Z. (2013). Impaired replication elongation in Tetrahymena mutants deficient in histone H3 Lys 27 monomethylation. Genes. Dev..

[56] Jacob Y., Bergamin E., Donoghue M.T., Mongeon V., LeBlanc C., Voigt P., Underwood C.J., Brunzelle J.S., Michaels S.D., Reinberg D. (2014). Selective methylation of histone H3 variant H3.1 regulates heterochromatin replication. Science.

[57] Davarinejad H., Huang Y.C., Mermaz B., LeBlanc C., Poulet A., Thomson G., Joly V., Munoz M., Arvanitis-Vigneault A., Valsakumar D. (2022). The histone H3.1 variant regulates TONSOKU-mediated DNA repair during replication. Science.

[58] Boehm E.M., Gildenberg M.S., Washington M.T. (2016). The Many Roles of PCNA in Eukaryotic DNA Replication. Enzymes.

[59] Raynaud C., Sozzani R., Glab N., Domenichini S., Perennes C., Cella R., Kondorosi E., Bergounioux C. (2006). Two cell-cycle regulated SET-domain proteins interact with proliferating cell nuclear antigen (PCNA) in Arabidopsis. Plant J..

[60] Baumbusch L.O., Thorstensen T., Krauss V., Fischer A., Naumann K., Assalkhou R., Schulz I., Reuter G., Aalen R.B. (2001). The *Arabidopsis thaliana* genome contains at least 29 active genes encoding SET domain proteins that can be assigned to four evolutionarily conserved classes. Nucleic. Acids Res..

[61] Springer N.M., Napoli C.A., Selinger D.A., Pandey R., Cone K.C., Chandler V.L., Kaeppler H.F., Kaeppler S.M. (2003). Comparative analysis of SET domain proteins in maize and Arabidopsis reveals multiple duplications preceding the divergence of monocots and dicots. Plant Physiol..

[62] Jacob Y., Feng S., LeBlanc C.A., Bernatavichute Y.V., Stroud H., Cokus S., Johnson L.M., Pellegrini M., Jacobsen S.E., Michaels S.D. (2009). ATXR5 and ATXR6 are H3K27 monomethyltransferases required for chromatin structure and gene silencing. Nat. Struct. Mol. Biol..

[63] Jacob Y., Michaels S.D. (2009). H3K27me1 is E(z) in animals, but not in plants. Epigenetics.

[64] Jacob Y., Stroud H., Leblanc C., Feng S., Zhuo L., Caro E., Hassel C., Gutierrez C., Michaels S.D., Jacobsen S.E. (2010). Regulation of heterochromatic DNA replication by histone H3 lysine 27 methyltransferases. Nature.

[65] Lindroth A.M., Shultis D., Jasencakova Z., Fuchs J., Johnson L., Schubert D., Patnaik D., Pradhan S., Goodrich J., Schubert I. (2004). Dual histone H3 methylation marks at lysines 9 and 27 required for interaction with CHROMOMETHYLASE3. Embo. J..

[66] Borg M., Jacob Y., Susaki D., LeBlanc C., Buendia D., Axelsson E., Kawashima T., Voigt P., Boavida L., Becker J. (2020). Targeted reprogramming of H3K27me3 resets epigenetic memory in plant paternal chromatin. Nat. Cell Biol..

[67] Jacob Y., Voigt P. (2018). In Vitro Assays to Measure Histone Methyltransferase Activity Using Different Chromatin Substrates. Methods Mol. Biol..

[68] Zhao X., Wang Y., Wang Y., Liu Y., Gao S. (2017). Histone methyltransferase TXR1 is required for both H3 and H3.3 lysine 27 methylation in the well-known ciliated protist Tetrahymena thermophila. Sci. China Life Sci..

[69] Cui B., Liu Y., Gorovsky M.A. (2006). Deposition and function of histone H3 variants in Tetrahymena thermophila. Mol. Cell Biol..

[70] Takeda S., Tadele Z., Hofmann I., Probst A.V., Angelis K.J., Kaya H., Araki T., Mengiste T., Mittelsten Scheid O., Shibahara K. (2004). BRU1, a novel link between responses to DNA damage and epigenetic gene silencing in Arabidopsis. Genes. Dev..

[71] Guyomarc’h S., Vernoux T., Traas J., Zhou D.X., Delarue M. (2004). MGOUN3, an Arabidopsis gene with TetratricoPeptide-Repeat-related motifs, regulates meristem cellular organization. J. Exp. Bot..

[72] Suzuki T., Inagaki S., Nakajima S., Akashi T., Ohto M.A., Kobayashi M., Seki M., Shinozaki K., Kato T., Tabata S. (2004). A novel Arabidopsis gene TONSOKU is required for proper cell arrangement in root and shoot apical meristems. Plant J..

[73] Duro E., Lundin C., Ask K., Sanchez-Pulido L., MacArtney T.J., Toth R., Ponting C.P., Groth A., Helleday T., Rouse J. (2010). Identification of the MMS22L-TONSL complex that promotes homologous recombination. Mol. Cell.

[74] O’Connell B.C., Adamson B., Lydeard J.R., Sowa M.E., Ciccia A., Bredemeyer A.L., Schlabach M., Gygi S.P., Elledge S.J., Harper J.W. (2010). A genome-wide camptothecin sensitivity screen identifies a mammalian MMS22L-NFKBIL2 complex required for genomic stability. Mol. Cell.

[75] O’Donnell L., Panier S., Wildenhain J., Tkach J.M., Al-Hakim A., Landry M.C., Escribano-Diaz C., Szilard R.K., Young J.T., Munro M. (2010). The MMS22L-TONSL complex mediates recovery from replication stress and homologous recombination. Mol. Cell.

[76] Piwko W., Olma M.H., Held M., Bianco J.N., Pedrioli P.G., Hofmann K., Pasero P., Gerlich D.W., Peter M. (2010). RNAi-based screening identifies the Mms22L-Nfkbil2 complex as a novel regulator of DNA replication in human cells. Embo. J..

[77] Campos E.I., Smits A.H., Kang Y.H., Landry S., Escobar T.M., Nayak S., Ueberheide B.M., Durocher D., Vermeulen M., Hurwitz J. (2015). Analysis of the Histone H3.1 Interactome: A Suitable Chaperone for the Right Event. Mol. Cell.

[78] Saredi G., Huang H., Hammond C.M., Alabert C., Bekker-Jensen S., Forne I., Reveron-Gomez N., Foster B.M., Mlejnkova L., Bartke T. (2016). H4K20me0 marks post-replicative chromatin and recruits the TONSL-MMS22L DNA repair complex. Nature.

[79] Jumper J., Evans R., Pritzel A., Green T., Figurnov M., Ronneberger O., Tunyasuvunakool K., Bates R., Zidek A., Potapenko A. (2021). Highly accurate protein structure prediction with AlphaFold. Nature.

[80] Piwko W., Mlejnkova L.J., Mutreja K., Ranjha L., Stafa D., Smirnov A., Brodersen M.M., Zellweger R., Sturzenegger A., Janscak P. (2016). The MMS22L-TONSL heterodimer directly promotes RAD51-dependent recombination upon replication stress. Embo. J..

[81] Huang T.H., Fowler F., Chen C.C., Shen Z.J., Sleckman B., Tyler J.K. (2018). The Histone Chaperones ASF1 and CAF-1 Promote MMS22L-TONSL-Mediated Rad51 Loading onto ssDNA during Homologous Recombination in Human Cells. Mol. Cell.

[82] Dong J., LeBlanc C., Poulet A., Mermaz B., Villarino G., Webb K.M., Joly V., Mendez J., Voigt P., Jacob Y. (2021). H3.1K27me1 maintains transcriptional silencing and genome stability by preventing GCN5-mediated histone acetylation. Plant Cell.

[83] Houston S.I., McManus K.J., Adams M.M., Sims J.K., Carpenter P.B., Hendzel M.J., Rice J.C. (2008). Catalytic function of the PR-Set7 histone H4 lysine 20 monomethyltransferase is essential for mitotic entry and genomic stability. J. Biol. Chem..

[84] Jorgensen S., Elvers I., Trelle M.B., Menzel T., Eskildsen M., Jensen O.N., Helleday T., Helin K., Sorensen C.S. (2007). The histone methyltransferase SET8 is required for S-phase progression. J. Cell Biol..

[85] Oda H., Okamoto I., Murphy N., Chu J., Price S.M., Shen M.M., Torres-Padilla M.E., Heard E., Reinberg D. (2009). Monomethylation of histone H4-lysine 20 is involved in chromosome structure and stability and is essential for mouse development. Mol. Cell Biol..

[86] Tardat M., Murr R., Herceg Z., Sardet C., Julien E. (2007). PR-Set7-dependent lysine methylation ensures genome replication and stability through S phase. J. Cell Biol..

[87] Fang J., Feng Q., Ketel C.S., Wang H., Cao R., Xia L., Erdjument-Bromage H., Tempst P., Simon J.A., Zhang Y. (2002). Purification and functional characterization of SET8, a nucleosomal histone H4-lysine 20-specific methyltransferase. Curr. Biol..

[88] Nishioka K., Rice J.C., Sarma K., Erdjument-Bromage H., Werner J., Wang Y., Chuikov S., Valenzuela P., Tempst P., Steward R. (2002). PR-Set7 is a nucleosome-specific methyltransferase that modifies lysine 20 of histone H4 and is associated with silent chromatin. Mol. Cell.

[89] Rice J.C., Nishioka K., Sarma K., Steward R., Reinberg D., Allis C.D. (2002). Mitotic-specific methylation of histone H4 Lys 20 follows increased PR-Set7 expression and its localization to mitotic chromosomes. Genes. Dev..

[90] Kuo A.J., Song J., Cheung P., Ishibe-Murakami S., Yamazoe S., Chen J.K., Patel D.J., Gozani O. (2012). The BAH domain of ORC1 links H4K20me2 to DNA replication licensing and Meier-Gorlin syndrome. Nature.

[91] Li S., Yang Z., Du X., Liu R., Wilkinson A.W., Gozani O., Jacobsen S.E., Patel D.J., Du J. (2016). Structural Basis for the Unique Multivalent Readout of Unmodified H3 Tail by Arabidopsis ORC1b BAH-PHD Cassette. Structure.

[92] Nakamura K., Saredi G., Becker J.R., Foster B.M., Nguyen N.V., Beyer T.E., Cesa L.C., Faull P.A., Lukauskas S., Frimurer T. (2019). H4K20me0 recognition by BRCA1-BARD1 directs homologous recombination to sister chromatids. Nat. Cell Biol..

[93] Alabert C., Bukowski-Wills J.C., Lee S.B., Kustatscher G., Nakamura K., de Lima Alves F., Menard P., Mejlvang J., Rappsilber J., Groth A. (2014). Nascent chromatin capture proteomics determines chromatin dynamics during DNA replication and identifies unknown fork components. Nat. Cell Biol..

[94] Ferrari K.J., Scelfo A., Jammula S., Cuomo A., Barozzi I., Stutzer A., Fischle W., Bonaldi T., Pasini D. (2014). Polycomb-dependent H3K27me1 and H3K27me2 regulate active transcription and enhancer fidelity. Mol. Cell.

[95] Jiang D., Berger F. (2017). DNA replication-coupled histone modification maintains Polycomb gene silencing in plants. Science.

[96] Pesavento J.J., Yang H., Kelleher N.L., Mizzen C.A. (2008). Certain and progressive methylation of histone H4 at lysine 20 during the cell cycle. Mol. Cell Biol..

[97] Huen M.S., Sy S.M., van Deursen J.M., Chen J. (2008). Direct interaction between SET8 and proliferating cell nuclear antigen couples H4-K20 methylation with DNA replication. J. Biol. Chem..

[98] Abbas T., Shibata E., Park J., Jha S., Karnani N., Dutta A. (2010). CRL4(Cdt2) regulates cell proliferation and histone gene expression by targeting PR-Set7/Set8 for degradation. Mol. Cell.

[99] Centore R.C., Havens C.G., Manning A.L., Li J.M., Flynn R.L., Tse A., Jin J., Dyson N.J., Walter J.C., Zou L. (2010). CRL4(Cdt2)-mediated destruction of the histone methyltransferase Set8 prevents premature chromatin compaction in S phase. Mol. Cell.

[100] Jorgensen S., Eskildsen M., Fugger K., Hansen L., Larsen M.S., Kousholt A.N., Syljuasen R.G., Trelle M.B., Jensen O.N., Helin K. (2011). SET8 is degraded via PCNA-coupled CRL4(CDT2) ubiquitylation in S phase and after UV irradiation. J. Cell Biol..

[101] Oda H., Hubner M.R., Beck D.B., Vermeulen M., Hurwitz J., Spector D.L., Reinberg D. (2010). Regulation of the histone H4 monomethylase PR-Set7 by CRL4(Cdt2)-mediated PCNA-dependent degradation during DNA damage. Mol. Cell.

[102] Tardat M., Brustel J., Kirsh O., Lefevbre C., Callanan M., Sardet C., Julien E. (2010). The histone H4 Lys 20 methyltransferase PR-Set7 regulates replication origins in mammalian cells. Nat. Cell Biol..

[103] Davarinejad H., Joshi M., Ait-Hamou N., Munro K., Couture J.F. (2019). ATXR5/6 Forms Alternative Protein Complexes with PCNA and the Nucleosome Core Particle. J. Mol. Biol..

[104] Brambati A., Barry R.M., Sfeir A. (2020). DNA polymerase theta (Poltheta)—An error-prone polymerase necessary for genome stability. Curr. Opin. Genet. Dev..

[105] Wang Q., Goldstein M., Alexander P., Wakeman T.P., Sun T., Feng J., Lou Z., Kastan M.B., Wang X.F. (2014). Rad17 recruits the MRE11-RAD50-NBS1 complex to regulate the cellular response to DNA double-strand breaks. Embo. J..

[106] Heitzeberg F., Chen I.P., Hartung F., Orel N., Angelis K.J., Puchta H. (2004). The Rad17 homologue of Arabidopsis is involved in the regulation of DNA damage repair and homologous recombination. Plant J..

[107] Ingouff M., Berger F. (2010). Histone3 variants in plants. Chromosoma.

[108] Filipescu D., Muller S., Almouzni G. (2014). Histone H3 variants and their chaperones during development and disease: Contributing to epigenetic control. Annu. Rev. Cell Dev. Biol..

[109] Cheng Z., Cheung P., Kuo A.J., Yukl E.T., Wilmot C.M., Gozani O., Patel D.J. (2014). A molecular threading mechanism underlies Jumonji lysine demethylase KDM2A regulation of methylated H3K36. Genes. Dev..

[110] Bowman A., Lercher L., Singh H.R., Zinne D., Timinszky G., Carlomagno T., Ladurner A.G. (2016). The histone chaperone sNASP binds a conserved peptide motif within the globular core of histone H3 through its TPR repeats. Nucleic. Acids Res..

[111] Zhang M., Liu H., Gao Y., Zhu Z., Chen Z., Zheng P., Xue L., Li J., Teng M., Niu L. (2016). Structural Insights into the Association of Hif1 with Histones H2A-H2B Dimer and H3-H4 Tetramer. Structure.

[112] Hong S., Cho Y.W., Yu L.R., Yu H., Veenstra T.D., Ge K. (2007). Identification of JmjC domain-containing UTX and JMJD3 as histone H3 lysine 27 demethylases. Proc. Natl. Acad. Sci. USA.

[113] Xu S., Wu J., Sun B., Zhong C., Ding J. (2011). Structural and biochemical studies of human lysine methyltransferase Smyd3 reveal the important functional roles of its post-SET and TPR domains and the regulation of its activity by DNA binding. Nucleic. Acids. Res..

[114] Burrage L.C., Reynolds J.J., Baratang N.V., Phillips J.B., Wegner J., McFarquhar A., Higgs M.R., Christiansen A.E., Lanza D.G., Seavitt J.R. (2019). Bi-allelic Variants in TONSL Cause SPONASTRIME Dysplasia and a Spectrum of Skeletal Dysplasia Phenotypes. Am. J. Hum. Genet..

[115] Chang H.R., Cho S.Y., Lee J.H., Lee E., Seo J., Lee H.R., Cavalcanti D.P., Makitie O., Valta H., Girisha K.M. (2019). Hypomorphic Mutations in TONSL Cause SPONASTRIME Dysplasia. Am. J. Hum. Genet..

[116] Micale L., Cialfi S., Fusco C., Cinque L., Castellana S., Biagini T., Talora C., Notarangelo A., Bisceglia L., Taruscio D. (2020). Novel TONSL variants cause SPONASTRIME dysplasia and associate with spontaneous chromosome breaks, defective cell proliferation and apoptosis. Hum. Mol. Genet..

[117] Feng W., Hale C.J., Over R.S., Cokus S.J., Jacobsen S.E., Michaels S.D. (2017). Large-scale heterochromatin remodeling linked to overreplication-associated DNA damage. Proc. Natl. Acad. Sci. USA.

[118] Hale C.J., Potok M.E., Lopez J., Do T., Liu A., Gallego-Bartolome J., Michaels S.D., Jacobsen S.E. (2016). Identification of Multiple Proteins Coupling Transcriptional Gene Silencing to Genome Stability in *Arabidopsis thaliana*. PLoS Genet..

[119] Ma Z., Castillo-Gonzalez C., Wang Z., Sun D., Hu X., Shen X., Potok M.E., Zhang X. (2018). Arabidopsis Serrate Coordinates Histone Methyltransferases ATXR5/6 and RNA Processing Factor RDR6 to Regulate Transposon Expression. Dev. Cell.

[120] Stroud H., Hale C.J., Feng S., Caro E., Jacob Y., Michaels S.D., Jacobsen S.E. (2012). DNA methyltransferases are required to induce heterochromatic re-replication in Arabidopsis. PLoS Genet..

[121] Potok M.E., Zhong Z., Picard C.L., Liu Q., Do T., Jacobsen C.E., Sakr O., Naranbaatar B., Thilakaratne R., Khnkoyan Z. (2022). The role of ATXR6 expression in modulating genome stability and transposable element repression in Arabidopsis. Proc. Natl. Acad. Sci. USA.

[122] Ohno Y., Narangajavana J., Yamamoto A., Hattori T., Kagaya Y., Paszkowski J., Gruissem W., Hennig L., Takeda S. (2011). Ectopic gene expression and organogenesis in Arabidopsis mutants missing BRU1 required for genome maintenance. Genetics.

[123] Picart-Picolo A., Grob S., Picault N., Franek M., Llauro C., Halter T., Maier T.R., Jobet E., Descombin J., Zhang P. (2020). Large tandem duplications affect gene expression, 3D organization, and plant-pathogen response. Genome. Res..

[124] Volk A., Crispino J.D. (2015). The role of the chromatin assembly complex (CAF-1) and its p60 subunit (CHAF1b) in homeostasis and disease. Biochim. Biophys. Acta.

[125] Schwartzentruber J., Korshunov A., Liu X.Y., Jones D.T., Pfaff E., Jacob K., Sturm D., Fontebasso A.M., Quang D.A., Tonjes M. (2012). Driver mutations in histone H3.3 and chromatin remodelling genes in paediatric glioblastoma. Nature.

[126] Wu G., Broniscer A., McEachron T.A., Lu C., Paugh B.S., Becksfort J., Qu C., Ding L., Huether R., Parker M. (2012). Somatic histone H3 alterations in pediatric diffuse intrinsic pontine gliomas and non-brainstem glioblastomas. Nat. Genet..

[127] Sarthy J.F., Meers M.P., Janssens D.H., Henikoff J.G., Feldman H., Paddison P.J., Lockwood C.M., Vitanza N.A., Olson J.M., Ahmad K. (2020). Histone deposition pathways determine the chromatin landscapes of H3.1 and H3.3 K27M oncohistones. eLife.

